# The potential mechanism of Aidi injection against neuroblastoma—an investigation based on network pharmacology analysis

**DOI:** 10.3389/fphar.2024.1310009

**Published:** 2024-01-19

**Authors:** Shuyang Dai, Yaoyao Gu, Yong Zhan, Jie Zhang, Lulu Xie, Yi Li, Yifei Lu, Ran Yang, Enqing Zhou, Deqian Chen, Songbin Liu, Shan Zheng, Zhaopeng Shi, Kuiran Dong, Rui Dong

**Affiliations:** ^1^ Shanghai Key Laboratory of Birth Defect, Department of Pediatric Surgery, Children’s Hospital of Fudan University, Shanghai, China; ^2^ Department of Anesthesiology, Huadong Hospital Affiliated to Fudan University, Shanghai, China; ^3^ Key Laboratory of Cell Differentiation and Apoptosis of the Chinese Ministry of Education, School of Medicine, Basic Medical Institute, Shanghai Jiao Tong University, Shanghai, China

**Keywords:** Aidi injection, neuroblastoma, network pharmacology, pharmacological mechanisms, molecular docking

## Abstract

**Background:** Aidi injection, a classic traditional Chinese medicine (TCM) formula, has been used on a broader scale in treating a variety of cancers. In this study, we aimed to explore the potential anti-tumor effects of Aidi injection in the treatment of neuroblastoma (NB) using network pharmacology (NP).

**Methods:** To elucidate the anti-NB mechanism of Aidi injection, an NP-based approach and molecular docking validation were employed. The compounds and target genes were collected from the Traditional Chinese Medicine Systems Pharmacology (TCMSP) database and Bioinformatics Analysis Tool for Molecular mechANism of Traditional Chinese Medicine (BATMAN-TCM) database. The protein–protein interaction network was constructed using the STRING database. clusterProfiler (R package) was utilized to annotate the bioinformatics of hub target genes. The gene survival analysis was performed on R2, a web-based genomic analysis application. iGEMDOCK was used for molecular docking validation, and GROMACS was utilized to validate molecular docking results. Furthermore, we investigated the anticancer effects of gomisin B and ginsenoside Rh2 on human NB cells using a cell viability assay. The Western blot assay was used to validate the protein levels of target genes in gomisin B- and ginsenoside Rh2-treated NB cells.

**Results:** A total of 2 critical compounds with 16 hub target genes were identified for treating NB. All 16 hub genes could potentially influence the survival of NB patients. The top three genes (EGFR, ESR1, and MAPK1) were considered the central hub genes from the drug–compound–hub target gene–pathway network. The endocrine resistance and estrogen signaling pathways were identified as the therapeutic pathways using the Kyoto Encyclopedia of Genes and Genomes (KEGG) analysis. Gomisin B and ginsenoside Rh2 showed a good binding ability to the target protein in molecular docking. The results of cell experiments showed the anti-NB effect of gomisin B and ginsenoside Rh2. In addition, the administration of gomisin B over-regulated the expression of ESR1 protein in MYCN-amplified NB cells.

**Conclusion:** In the present study, we investigated the potential pharmacological mechanisms of Aidi against NB and revealed the anti-NB effect of gomisin B, providing clinical evidence of Aidi in treating NB and establishing baselines for further research.

## 1 Introduction

Neuroblastoma (NB) arises from neuroepithelial cells during the neural crest migration to the sympathetic nervous system and is noted as the most common extra-cranial solid tumor in children (Maris, 2010). Recent statistics indicate that the mortality rates of NB range 0.85–1.1 cases per 100,000 children under the age of 15, accounting for 15% of all pediatric deaths caused by cancers (Weinstein et al., 2003). By advancing and refining conventional treatment approaches like chemotherapy, radiotherapy, and surgery, patients with low- and intermediate-risk NB have experienced favorable outcomes, boasting an impressive 5-year survival rate (Castel et al., 2007). Nevertheless, those diagnosed with high-risk neuroblastoma (HR-NB) still grapple with a less favorable prognosis, affecting approximately 60% of cases. Notably, MYCN amplification is prevalent in 20%–30% of NB patients, and the overall survival rate for this subgroup remains below 50% (Tsubota and Kadomatsu, 2018).

Numerous studies have shown the anti-tumor ability of traditional Chinese medicine (TCM) (Li and Zhang, 2008). Aidi injection, a traditional Chinese anti-tumor herbal preparation developed and manufactured by Guizhou Ebay Pharmaceutical Co., Ltd. in China, is used to treat a variety of cancers, such as lung cancer, gastrointestinal cancer, liver cancer, pancreatic cancer, and malignant lymphoma (Wang et al., 2018; Xu et al., 2011; Jiancheng et al., 2015; Mulati et al., 2014; Wang et al., 2022). In 2021, a new type of Aidi injection was invented and passed through patent licensing, which was initially designed for the NB treatment (AIDI, 2021). The main practical components of the new type of Aidi injection are Ban Mao (Mylabri [BM]), Ci Wu Jia (Radix Acanthopanax Senticosus [CWJ]), Huang Qi (Radix Astragali [HQ]), and Ren Shen (Radix Ginseng [RS]).

The Traditional Chinese Medicine Systems Pharmacology (TCMSP) database is an open-source platform used for the discovery of natural anti-tumor small-molecule compounds in TCM prescriptions (Ru et al., 2014). Network pharmacology (NP) is a promising and systematic approach that utilizes molecular networks to identify connections between various diseases and TCM formulas (Luo et al., 2019). Consequently, it enables the determination of the pharmacological mechanisms underlying the activity of active compounds.

**FIGURE 1:**
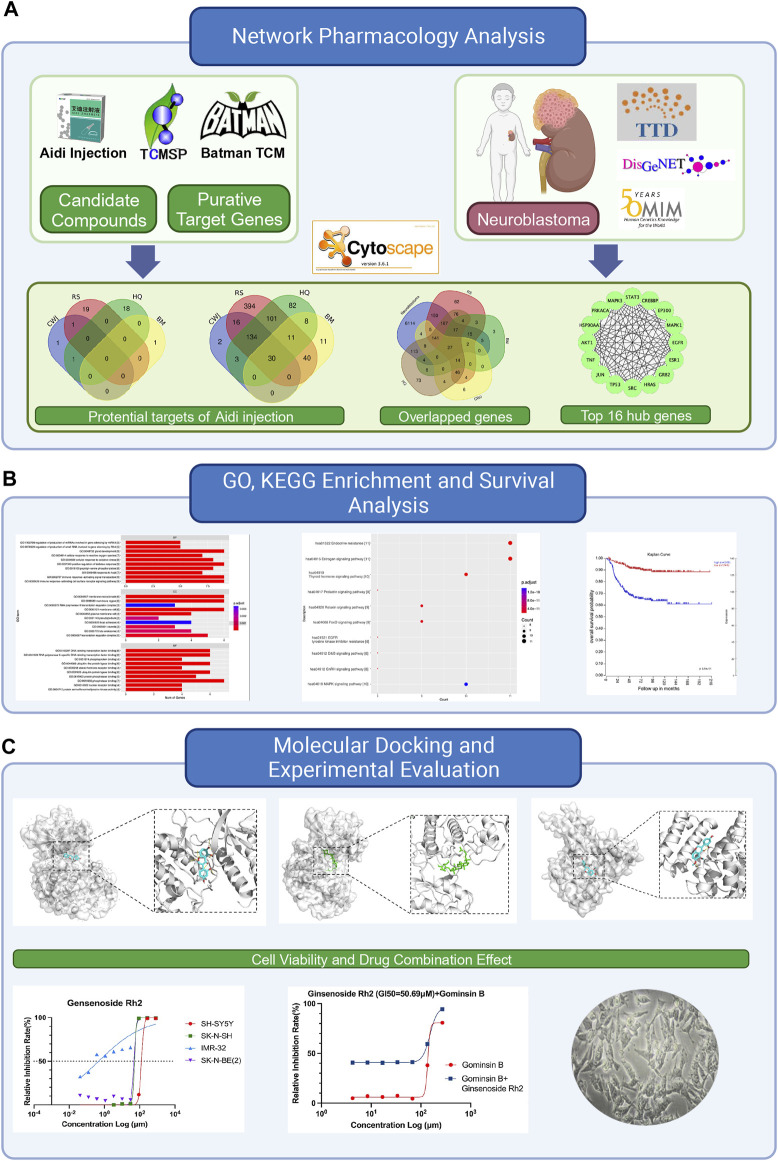
Flowchart of this study to explore the potential anti-tumor effects of Aidi in the treatment of neuroblastoma based on network pharmacology. **(A)** Details of the network pharmacology analysis of Aidi injection, involving the identification of potential targets, overlapped genes, and top hub genes. **(B)** Exploration of Gene Ontology (GO), Kyoto Encyclopedia of Genes and Genomes (KEGG), and survival analysis. **(C)** Flowchart of the molecular docking process and experimental evaluation of the key compounds of Aidi injection.

This study aims to discover the effective compounds and pharmacological mechanisms of Aidi injection in the treatment of NB through the NP strategy (Figure 1).

## 2 Methods

### 2.1 Database establishment

The drug compounds and target genes of four main agents (Mylabri [BM], Radix Acanthopanax Senticosus [CWJ], Radix Astragali [HQ], and Radix Ginseng [RS]) in Aidi injection were initially collected from the Traditional Chinese Medicine Systems Pharmacology database (TCMSP, http://lsp.nwu.edu.cn/tcmsp.php) and the Bioinformatics Analysis Tool for Molecular mechANism of Traditional Chinese Medicine database (BATMAN-TCM, http://bionet.ncpsb.org/batman-tcm/index.php) (Liu et al., 2016). The TCMSP database comprises 499 Chinese herbs with 29,384 ingredients, 3,311 targets, and 837 related diseases. The oral bioavailability (OB) is set over or equal to 30%, and the drug-likeness (DL) is greater than 0.18 for different compounds based on the instructions of TCMSP (Li and Zhang, 2013). To validate the target gene symbols obtained from TCMSP, we cross-referenced them using the UniProt database (https://www.UniProt.org/), specifically selecting the species “*Homo sapiens*” (human) (The UniProt Consortium, 2023). The score of each target gene of the compound can be obtained using BATMAN-TCM, which shows the correlation with the target gene. The correlation score of the target gene above the mean score of all target genes of Aidi injection should be enrolled in this study (Luo et al., 2019). The final database of drug compounds and target genes was constructed based on the combination of compounds and target genes from TCMSP and BATMAN-TCM.

### 2.2 Identification of putative target genes for NB

After the comprehensive mining in DisGeNET (v 7.0), Online Mendelian Inheritance in Man (OMIM), and the Therapeutic Target Database (TTD), the putative target genes of NB were collected for further study. DisGeNET, a discovery platform, contains comprehensive data on multiple targets of genes and their associated human diseases (Piñero et al., 2016). OMIM provides disease-related genetic information based on the published research and contains over 15,000 genes (Hamosh et al., 2004). TTD provides basic information, corresponding target genes, and related pathways of 34,019 drugs (Zhou et al., 2021).

### 2.3 Construction of the protein–protein interaction network

The intersection set of target genes of Aidi in the treatment of NB was identified as a co-occurrence of putative target genes of Aidi and NB. In order to retrieve the interacting gene in the intersection set of putative target genes, the STRING database (11.0, https://string-db.org/) was used to construct the protein–protein interaction (PPI) network (Szklarczyk et al., 2021; Zhou et al., 2021). To ensure the reliability of the results, the interaction score in the STRING database was set to be equal to or greater than 0.9. This stringent threshold helps focus on high-confidence PPIs, thereby preserving the robustness of the findings. Then, Cytoscape (version 3.71) was used to visualize and analyze the PPI networks. cytoHubba from Cytoscape was used to further identify the hub genes. The top 16 genes based on the maximum neighborhood component (MCC) were considered the hub target genes (Zhou et al., 2021).

### 2.4 Bioinformatic annotations of hub target genes

We utilized clusterProfiler, a package available in R software version 3.6.1, to perform Gene Ontology (GO) and Kyoto Encyclopedia of Genes and Genomes (KEGG) pathway enrichment analyses for the hub target genes (Ashburner et al., 2000; Kanehisa et al., 2016; Mi et al., 2018; Consortium et al., 2020). Molecular function (MF), biological process (BP), and cellular component (CC) were identified in the GO analysis. A *p*-value ≤0.01 was considered statistically significant (Gene Ontology Consortium, 2009).

### 2.5 Identification of clinical significance of hub genes

R2 (http://r2.amc.nl http://r2platform.com) is a web-based genomic analysis and visualization application developed by Jan Koster at the Department of Oncogenomics at the Academic Medical Center (AMC) Amsterdam, Netherlands (Koster et al., 2019). This platform provides researchers with a user-friendly interface to perform various genomic analyses and efficiently visualize genomic data. The R2 platform consists of two main components: a publicly accessible database and a set of tools that can mine the database. Two main NB patient databases were accessible from GEO (https://www.ncbi.nlm.nih.gov/geo/), namely, GSE49710 (n = 498) and GSE49711 (n = 498) through the R2 platform. These databases include both microarray data and RNA-Seq, allowing for an investigation of overall survival probability using the Kaplan–Meier scan. We analyzed the correlation between the expression levels of hub genes and overall survival probability using the Kaplan–Meier method.

Then, we normalized the gene expression matrix using the DESeq2 package from R software. Next, we performed the survival analysis to investigate the influence of the hub genes on the survival of NB patients. Hub genes with statistical significance in both the Cox regression and log-rank tests were provided. Survival curves of the hub genes were plotted using the R2 platform. The expression pattern of hub genes that impact NB patient survival was determined once the hub genes were discovered.

### 2.6 Drug–compound–hub target gene–pathway network construction

Cytoscape 3.71 was used to generate the drug–compound–hub target gene–pathway network from the identified hub genes and KEGG pathways. cytoHubba was used to filter out significant hub genes and key compounds in this network. A total of 16 compound nodes with the highest value in all nodes were regarded as vital compounds of Aidi for NB treatment. Additionally, hub genes with the highest degree values were identified as important target genes for Aidi in the treatment of NB.

### 2.7 Validation of key compound–hub target gene interactions

Additionally, the molecular docking method was used to confirm the interaction between the key chemical and the hub target gene. The RCSB PDB database (https://www.rcsb.org/) was used to retrieve the 3D structures of proteins expressed by hub target genes (Orm et al., 1996). Furthermore, the 3D structures of the rigor Aidi compounds were collected in SDF format from the PubChem database (https://pubchem.ncbi.nlm.nih.gov/) and then converted into PDB format using PyMOL software (version 2.2) (Bramucci et al., 2012; Kim et al., 2020). Next, the hydrone and extra ligands of the hub target proteins were removed using PyMOL software. We used iGEMDOCK version 2.1 to perform the molecular docking (set population size = 20, generations = 70, and number of solutions = 2.) (Hsu et al., 2011). The total energy comprises electrostatic, steric, and hydrogen bonding potentials. The lower the total energy, the more stable the construction.

### 2.8 Molecular dynamic simulation

The resulting protein was isolated from the small-molecule ligand. Subsequently, the force field parameters for the small molecule were generated using the antechamber tool from AmberTools software. These parameters were then converted into GROMACS-compatible force field files utilizing the acpype software tool. Specifically, the small molecule was assigned the GAFF, while the protein was assigned the AMBER14SB force field, along with the TIP3P water model. The protein and small-molecule ligand files were amalgamated to construct a simulation system for the complex.

Molecular dynamic (MD) simulations were conducted within the GROMACS 2022 program, under constant temperature and pressure and periodic boundary conditions. Throughout the MD simulation process, hydrogen bonds were rigorously constrained using the LINCS algorithm, with an integration time step of 2 fs. Electrostatic interactions were computed utilizing the particle mesh Ewald (PME) method, with a cutoff distance of 1.2 nm. Non-bonded interactions were truncated at 10 Å and updated at intervals of every 10 time steps. Temperature was controlled at 298 K using the V-rescale temperature coupling method, while pressure was maintained at 1 bar using the Berendsen method.

The simulation commenced with constant number, volume, and temperature (NVT) and constant number, pressure, and temperature (NPT) equilibration runs, each lasting 100 ps at 298 K. Subsequently, a 100 ns MD simulation of the complex system was executed, with configurations saved every 10 ps. Following the completion of the simulation, the resultant trajectories were subjected to a comprehensive analysis. The binding free energy of the complex was calculated using the g_mmpbsa program, employing the MMPBSA methodology.

### 2.9 Cell cultures, drugs, and reagents

The human NB cell lines (SK-N-SH, SH-SY5Y, SK-N-BE (2), and IMR-32) were obtained from the National Collection of Authenticated Cell Cultures in Shanghai, China.

SK-N-SH and IMR-32 cells were cultured in Dulbecco’s modified Eagle medium (DMEM) containing 10% fetal bovine serum (FBS) (Gibco) and 1% penicillin–streptomycin mixture (Sigma-Aldrich). SK-N-BE (2) cells were cultured in DMEM/F12 (1:1) with 10% FBS and 1% penicillin–streptomycin mixture (Sigma-Aldrich). SH-SY5Y cells were cultured in a mixture of MEM (44.5%) and Ham’s F12 (44.5%), supplemented with 10% FBS (Gibco), 1% non-essential amino acids (NEAAs) (Gibco), and 1% penicillin–streptomycin mixture (Sigma-Aldrich). All cells were maintained in an incubator at 37°C with a humidified atmosphere containing 5% CO_2_.

Gomisin B and ginsenoside Rh2 chemical reagents were procured from MedChemExpress. Gomisin B (purity ≥99.9%) and ginsenoside Rh2 (purity ≥99.9%) were dissolved in dimethyl sulfoxide (DMSO). To ensure minimal impact on cells, the final concentration of DMSO in the cell culture medium was maintained below 0.1%.

Cell viability was assessed using the CellCounting-Lite 2.0 Luminescent Cell Viability Assay (Vazyme, DD1101-03) and measured using a multimode microplate detection system (PerkinElmer EnVision 2015). Aidi injection was donated by Guizhou Ebay Pharmaceutical Co., Ltd.

### 2.10 Experiments assessing the effects of Aidi injection, gomisin B, and ginsenoside Rh2 on neuroblastoma cells

A suspension of 50,000 cells/mL of SK-N-SH, SH-SY5Y, SK-N-BE (2), and IMR-32 cells was added to each well of a 96-well plate. The 96-well plates were then incubated in a humidified 5% CO_2_ incubator at 37°C for 24 h. At this point, the cell medium was removed and replaced with a medium containing either Aidi injection, gomisin B, or ginsenoside Rh2 or a mixture of gomisin B and ginsenoside Rh2 dissolved in 1% DMSO. The cells were cultured in a medium for 48 h.

### 2.11 Luminescent cell viability assay

Cell proliferation was assessed using the CellCounting-Lite 2.0 Luminescent Cell Viability Assay. After incubation of the cells, 100 μl of CellCounting-Lite reagent was added to each well and allowed to set at room temperature for 10 min to stabilize the luminescent signal. Luminescent signals were then detected using a multifunctional enzyme marker (PerkinElmer EnVision 2015).

### 2.12 Western blot analysis

Proteins were extracted from cells after 48 h of incubation with the corresponding IC_50_ values of gomisin B, ginsenoside Rh2, or 1% DMSO (used as a negative control). RIPA Lysis and Extraction Buffer (Thermo Fisher Scientific, United States) containing 100× Halt™ Protease and Phosphatase Inhibitor Single-Use Cocktail (Thermo Fisher Scientific, United States) was utilized for protein extraction. Protein concentrations were determined using the BCA assay (Beyotime, Shanghai, China). The sample proteins were loaded and separated by electrophoresis using 4%–20% precast protein improve gels. Following wet transfer using 0.45-μm PVDF membranes, the membranes were blocked and then incubated with specific primary antibodies against EGFR, MAPK1, and GAPDH overnight at 4°C. Subsequently, an additional hour of incubation with HRP-conjugated anti-rabbit IgG was performed at room temperature. Finally, enhanced chemiluminescence using SuperSignal West Pico PLUS (Thermo Fisher Scientific) was employed to visualize the bands. Image acquisition was conducted using Image Lab software.

### 2.13 Data analysis

The results were expressed as relevant inhibition rates. GI_50_ values were determined by analyzing regression lines, assuming a linear response. F-tests were employed to assess significant differences between the slopes of the regression lines, assuming that the data followed a straight line. To determine the significance of the results obtained from cells exposed to different pteridine preparations or concentrations, one-way ANOVA and Student’s t-test were utilized. Description and symbols of synergism or antagonism in drug combination studies were analyzed using the CI method described by Chou and Talalay (1984).

## 3 Results

### 3.1 Active compounds and putative target genes of Aidi

**FIGURE 2:**
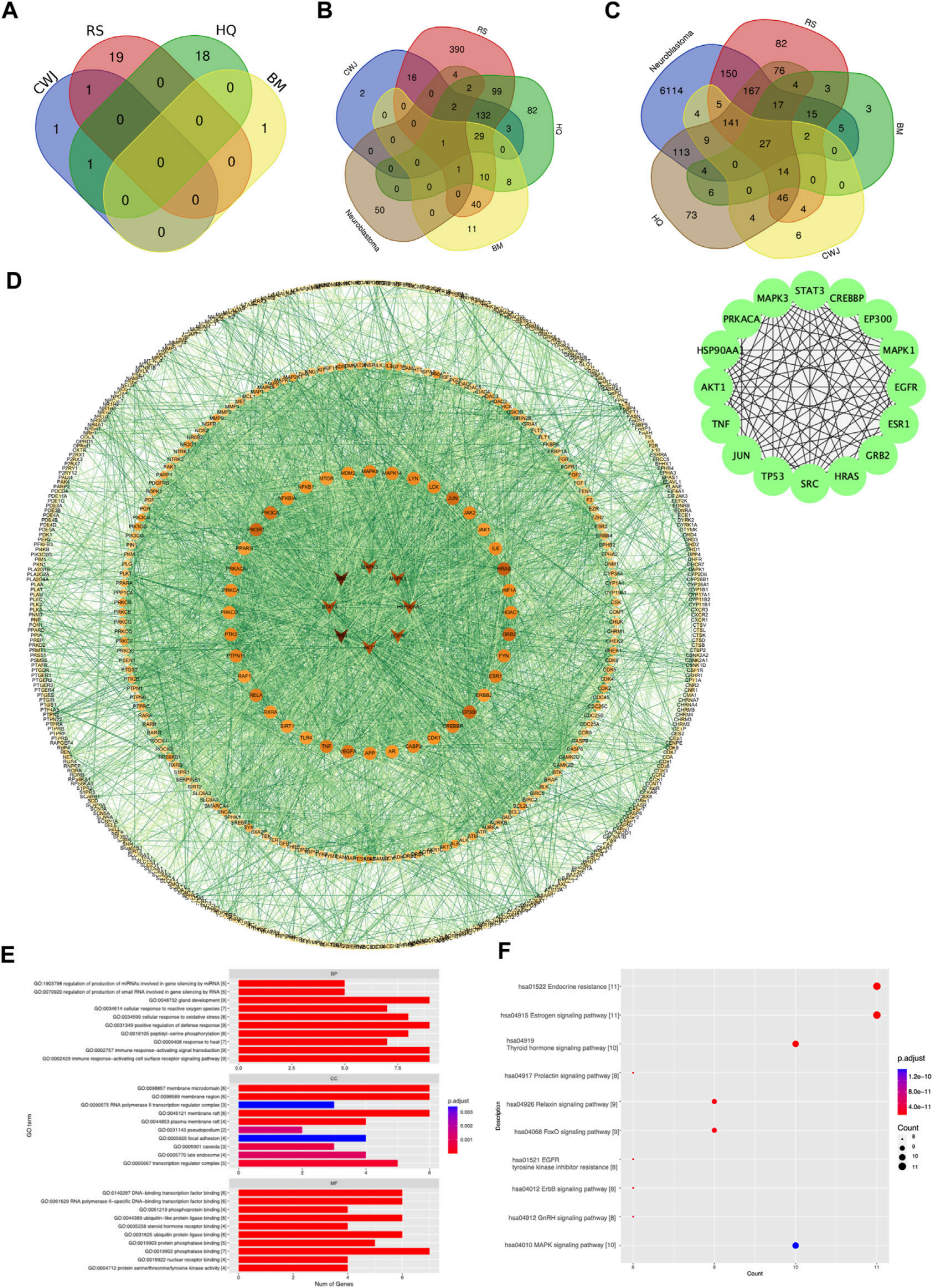
Venn diagram of the number of various compounds and target gene distribution. **(A)** Details of distribution numbers of active compounds in Aidi. The violet oval represents the identified CWJ compounds. The pink oval represents the identified RS compounds. The viridis and citrine ovals represent the HQ and BM compounds, respectively. **(B)** Details of different putative target genes in Aidi. The violet and pink ovals represent the identified CWJ and RS targets, respectively. The viridis oval represents the identified HQ target genes. The citrine oval represents the BM targets. **(C)** Venn diagram of the number relationship between the putative target genes of Aidi and neuroblastoma. **(D)** PPI network of target genes shared between Aidi and neuroblastoma. **(E)** Main GO terms enriched by hub target genes. **(F)** Main KEGG terms enriched by hub target genes.

A total of 41 active compounds were collected from the four Chinese herbal medications present in Aidi through the TCMSP and BATMAN-TCM databases. The total number of compounds in each Chinese medication containing Aidi and the number of compounds that overlapped are shown in Figure 2A.

In addition, a total of 829 putative target genes for Aidi were gathered. The numbers of putative target genes of BM, CWJ, HQ, and RS drugs were 100, 185, 369, and 726, respectively. Our results showed that 30 putative target genes overlapped in the four Chinese herbal medicines in Aidi injection. There were 49 overlapped putative target genes between HQ and BM, 246 overlapped putative target genes between RS and HQ, 180 overlapped putative target genes between CWJ and RS, 81 overlapped putative target genes between BM and RS, 30 overlapped putative target genes between CWJ and BM, and 167 overlapped putative target genes between CWJ and HQ. There were 164 overlapped putative target genes between CWJ, RS, and HQ, 30 overlapped putative target genes between CWJ, HQ, and BM, 41 overlapped putative target genes between BM, HQ, and RS, and 30 overlapped putative target genes between CWJ, HQ, and BM. The presence of common compounds and target genes among these four Chinese medicines indicated that they might have synergistic therapeutic effects during the treatment course.

### 3.2 Putative target genes between neuroblastoma and Aidi

The number of probable NB target genes that were obtained from the DisGeNET, OMIM, and GeneCards databases was 2,508, 193, and 6,179. Following the elimination of redundancy, a total of 6,773 unique potential target genes were identified and confirmed. It was discovered that there were 659 shared target genes between Aidi and NB when the data from putative target genes of the two cancers were combined. These common potential targets might play a significant role in the treatment of NB by Aidi injection (Figure 2C).

### 3.3 Analysis of the PPI network

To acquire the interactions of proteins, a total of 659 common putative target genes were enrolled in STRING 11.0. Cytoscape v.3.71 was used to form the PPI network, and STRING 11.0 was used to obtain the interactions of proteins (Figure 2D).

As a result, the PPI network contained 620 nodes and 5,129 edges. Ultimately, using cytoHubba as the selective method, 16 nodes were selected as hub nodes. Since these genes play an important role in the PPI network, all these 16 hub genes were identified as the hub target genes for Aidi to treat NB.

### 3.4 Results of bioinformatic annotation

Through GO analysis, a total of 1,209 GO terms were defined, of which 1,139 were BP, 11 were CC, and 59 were MF terms. The top 10 terms of BP, CC, and MF with adjusted *p*-values were presented eventually (Figure 2E). The major BPs included gland development, positive regulation of defense response, immune response-activating signal transduction, and the immune response-activating cell surface receptor signaling pathway. The major CC terms included the membrane microdomain, membrane region, and membrane raft. The major MF term included phosphatase binding. Regarding KEGG analysis, a total of 194 pathways were recognized and the top 10 KEGG pathways with significantly adjusted *p*-values were presented. The results of the KEGG enrichment analysis indicated that the main pathways of the hub genes against NB focused on the endocrine resistance and estrogen signaling pathway (Figure 2F). Therefore, Aidi might treat NB via the above GO terms and pathways.

### 3.5 Clinical significance of hub target genes in neuroblastoma

**FIGURE 3:**
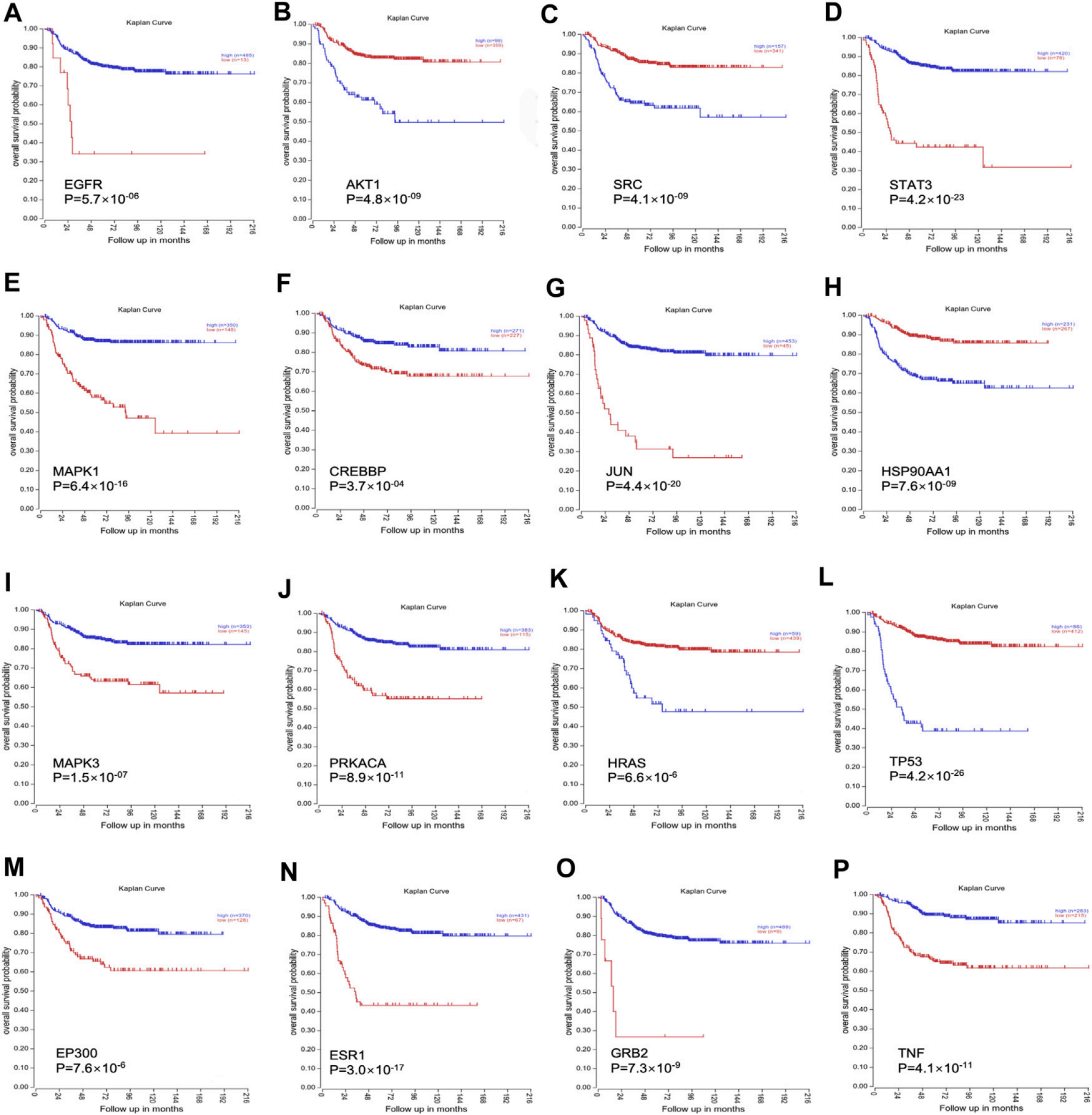
The clinical significance of all 16 genes was validated through the R2 platform. **(A)**. Overall survival probability of EGFR; **(B)**. Overall survival probability of AKT1; **(C)**. Overall survival probability of SRC; **(D)**. Overall survival probability of STAT3; **(E)**. Overall survival probability of MAPK1; **(F)**. Overall survival probability of CREBBP; **(G)**: Overall survival probability of JUN; **(H)**. Overall survival probability of HSP90AA1; **(I)**. Overall survival probability of MAPK3; **(J)**. Overall survival probability of PRKACA; **(K)**. Overall survival probability of HRAS; **(L)**. Overall survival probability of TP53; **(M)**. Overall survival probability of EP300; **(N)**. Overall survival probability of ESR1; **(O)**. Overall survival probability of GRB2; **(P)**. Overall survival probability of TNF.

RNA-Seq data of 498 NB patients were obtained from the R2 platform, which was suitable for defining the correlation between overall survival and gene expression in NB datasets. All 16 genes showed a strong correlation with the overall survival of NB, which could influence the survival of NB patients (Figure 3).

### 3.6 Analysis of the drug–compound–hub target gene–pathway network

**FIGURE 4:**
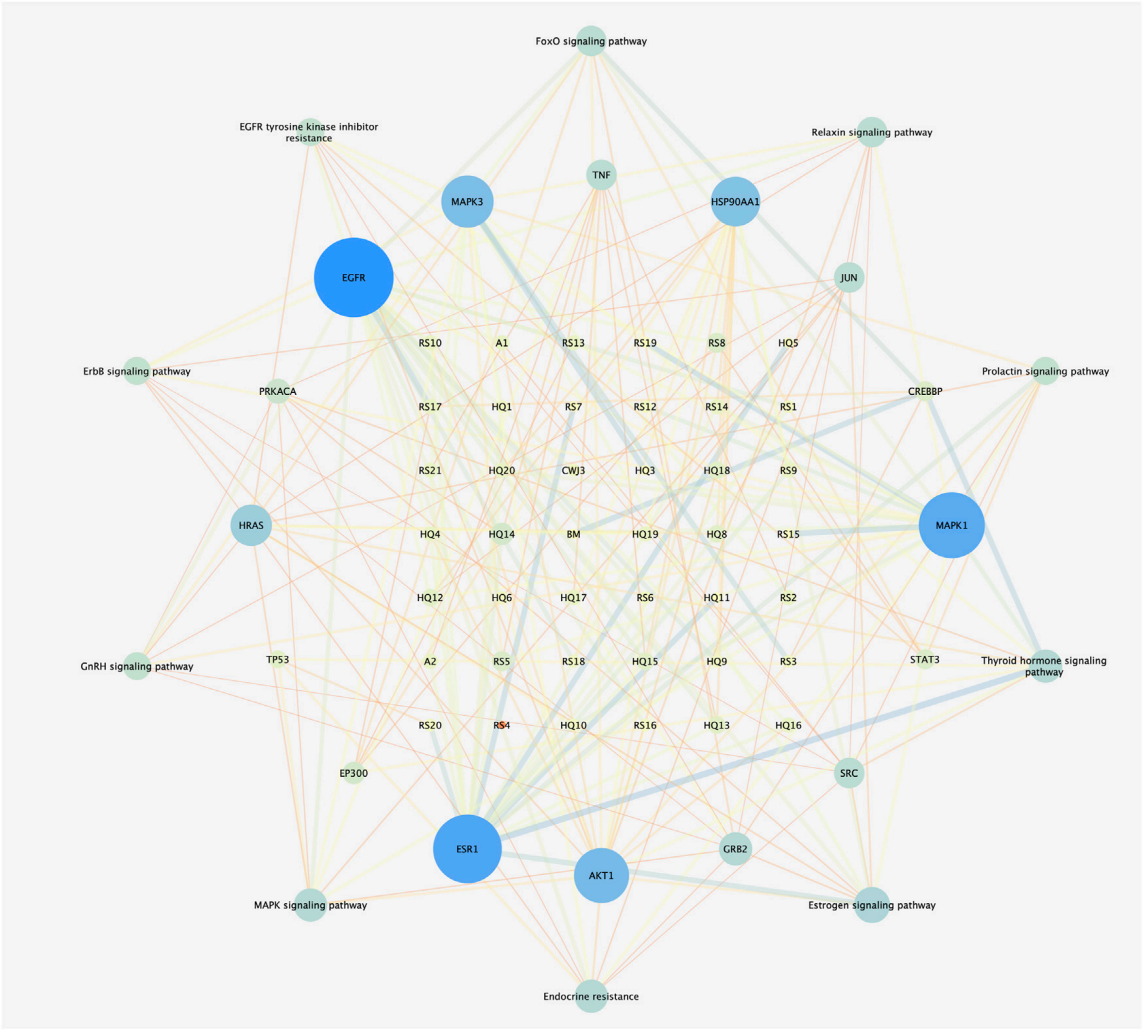
Interaction network of drug–compound–hub target gene–pathway.

The drug–compound–hub target gene–pathway network was constructed to find the major hub genes and the rigor compounds of Aidi for NB treatment. This network includes 68 nodes and 200 edges (Figure 4). The number of nodes and edges was closely related to their degree of contribution to the network. The top three genes with the highest value were considered to be the major hub genes, namely, EGFR (degree: 27), ESR1 (degree: 23), and MAPK1 (degree: 22). Meanwhile, the top compounds with the highest degree value were regarded as rigor compounds of Aidi, which included isoflavanone (HQ14, Mol000398), gomisin B (RS5, Mol005357), and ginsenoside Rh2 (RS8, Mol005344). The details of the rigor compounds of Aidi are shown in Table 1.

**TABLE 1:** Key compounds of Aidi for neuroblastoma.

Compound	Molecular structural formula	Degree	Closeness	Betweeness	Drug source	Mol ID	CAS code
Isoflavanone	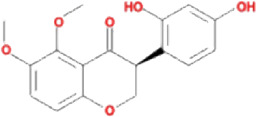	6	33.8333	167.0197	HQ	MOL000398	574-12-9
Gomisin B	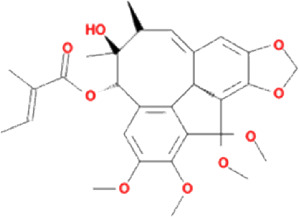	6	35.44	142.41201	RS	MOL005357	58546-55-7
Ginsenoside Rh2	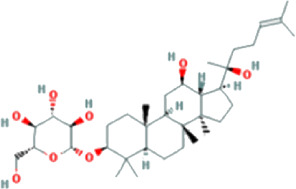	6	33.08333	129.34328	RS	MOL005344	78214-33-2

### 3.7 Results of molecular docking validation

**FIGURE 5:**
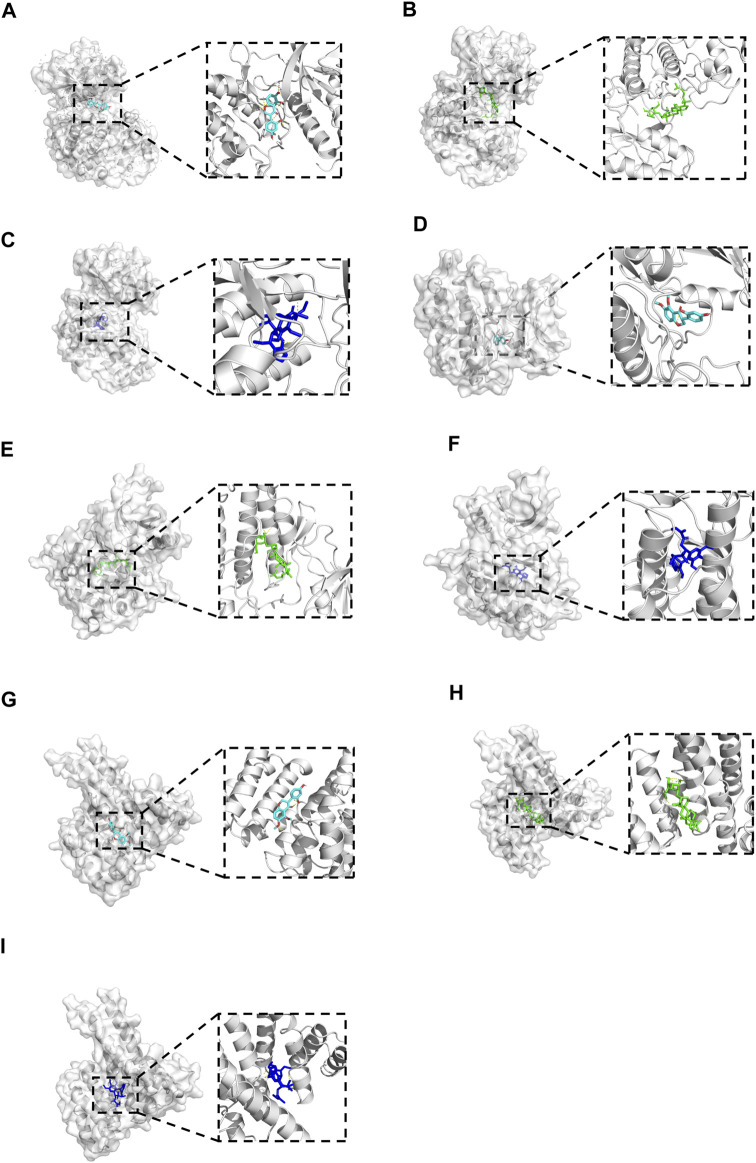
The 3D conformations of Isoflavanone, Gomisin B, and Ginsenoside Rh2 and hub target proteins. **(A-C)**: shows the Isoflavanone, Gominsin B and Ginsenoside rh2 is located in the binding pocket of MAPK1. **(D-F)**: shows the Isoflavanone, Gominsin B and Ginsenoside rh2 is located in the binding pocket of EGFR. **(G-I)**: shows the Isoflavanone, Gominsin B and Ginsenoside rh2 is located in the binding pocket of ESR1.

Molecular docking was performed between the key compounds and target protein expressed by the hub genes, which had an impact on NB patients’ survival. The docking total energy was calculated via iGEMDOCK, and the results are shown in Table 2. The docking structure of compounds and proteins is shown in Figure 5. The average total energy values of isoflavanone, gomisin B, and ginsenoside Rh2 were −89.9651, −102.5431, and −103.3466, respectively. Hence, gomisin B and ginsenoside Rh2 had the best binding interactions between the target proteins expressed by the hub genes related to the survival of NB patients.

**TABLE 2:** Results of molecular docking between key compounds and hub target proteins.

Key compound	Hub target protein		
	Docking total energy (kcal/mol)		
	MAPK1	EGFR	ESR1
Isoflavanone	−77.9194	−96.5158	−88.69
Gomisin B	−84.9449	−102.72	−126.5
Ginsenoside Rh2	−88.5183	−107.801	−119.35

### 3.8 Results of RMSD, RMSF, and stability analysis of small-molecule-binding proteins in molecular dynamic simulation

**FIGURE 6:**
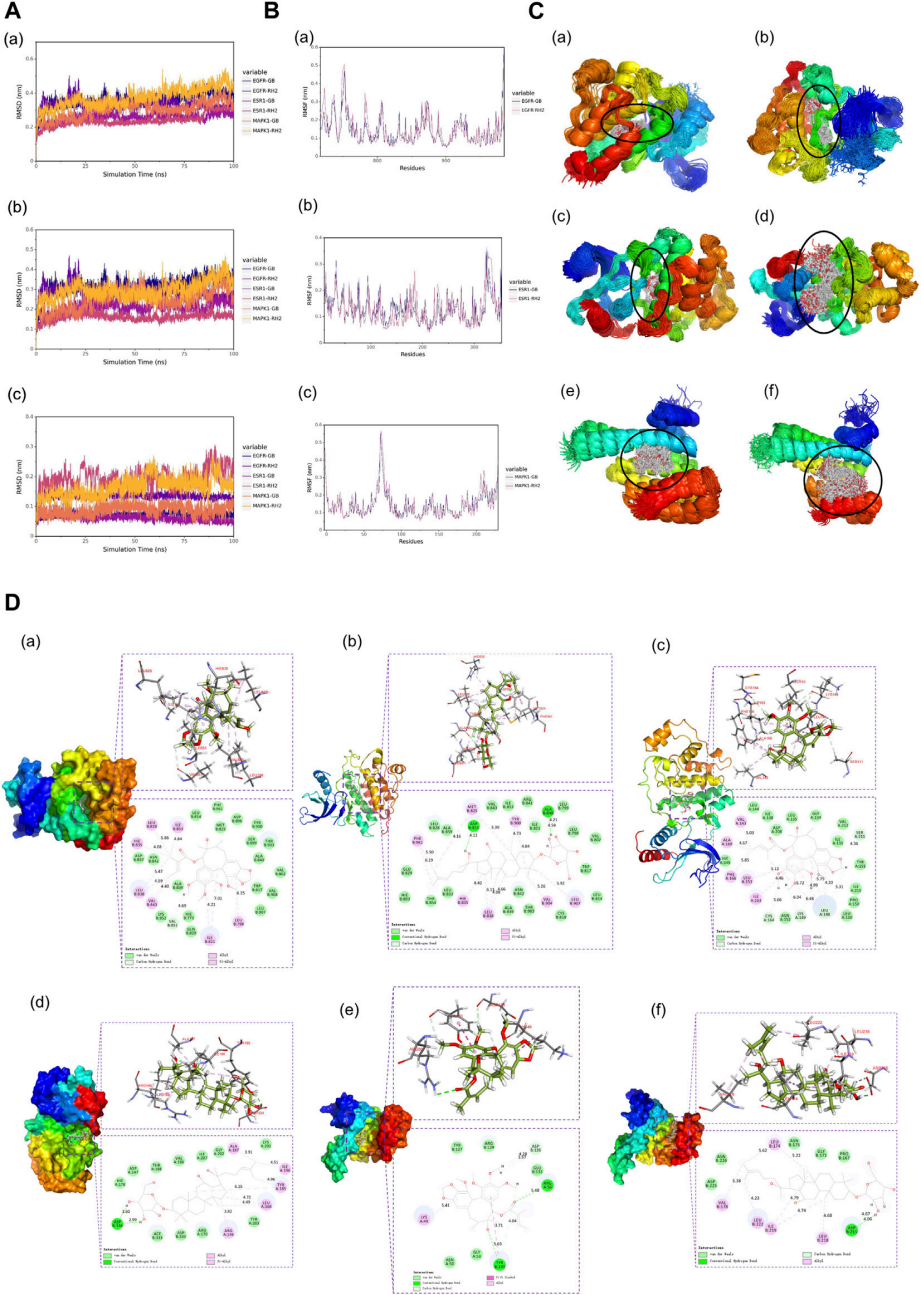
**(A)** RMSD analysis of each complex **(a)**, each protein **(b)**, and each small molecule **(c)**. **(B)** Root mean square fluctuation (RMSF) analysis of each protein and small molecule: **(a)** EGFR, **(b)** ESR1, and **(c)** MAPK1. **(C)** Simulated conformation of small molecule and protein: **(a)** EGFR-GB, **(b)** EGFR-RH2, **(c)** ESR1-GB, **(d)** ESR1-RH2, **(e)** MAPK1-GB, and **(f)** MAPK1-RH2. **(D)** Structure analysis: **(a)** EGFR-GB, **(b)** EGFR-RH2, **(c)** ESR1-GB, **(d)** ESR1-RH2, **(e)** MAPK1-GB, and **(f)** MAPK1-RH2.

Root mean square deviation (RMSD) is an essential factor to evaluate the stability of the system. As can be seen from Figure 6A, RMSD of proteins and complexes in each group was relatively stable, and RMSD of small molecules fluctuated within a certain range, indicating that the complexes in each group maintained a stable state. Root mean square fluctuation (RMSF) can indicate the amount of flexibility of amino acid residues in a protein. During simulation, the complex showed good stability (Figure 6B). It can be seen from Figure 6C that when small molecules were combined with the simulated conformation of proteins, the conformation of small molecules in each complex was near the initial binding site, and the degree of conformation was high, indicating that small molecules were always bound to the initial binding site of proteins during the simulation process. The results of small-molecule-binding site analysis showed that the binding of proteins to small molecules is stable. Furthermore, we showed the stable conformation of the simulated structure.

### 3.9 Results of the effects of Aidi injection, gomisin B, and ginsenoside Rh2 on viability

**FIGURE 7:**
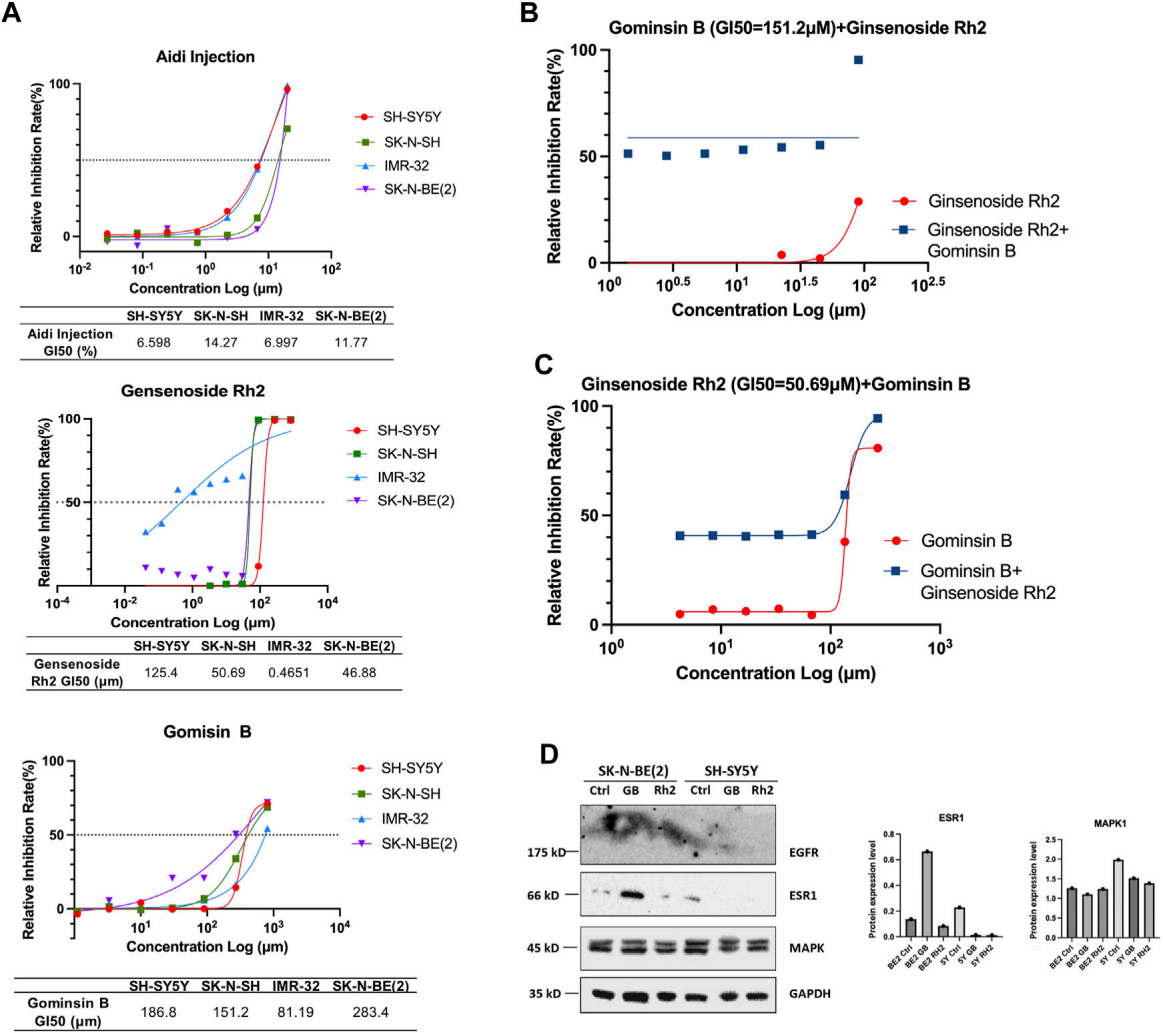
**(A)** Growth inhibition (GI_50_) of Aidi injection, ginsenoside Rh2, and gomisin B in four different neuroblastoma cell lines. The GI_50_ (%) values of Aidi injection are 6.598, 14.27, 6.997, and 11.77 in SH-SY5Y, SK-N-SH, IMR-32, and SK-N-BE (2), respectively. The GI_50_ (μM) values of Rh2 are 125.4, 50.69, 0.4651, and 46.88 in SH-SY5Y, SK-N-SH, IMR-32, and SK-N-BE (2), respectively. The GI50 (μM) values of gomisin B are 186.8, 151.2, 81.19, and 283.4 in SH-SY5Y, SK-N-SH, IMR-32, and SK-N-BE (2), respectively. **(B)** The synergism, as indicated by a combination index of 0.43, was observed only when the GI_50_ of gomisin B was 151.2 μM, and the concentration of ginsenoside Rh2 reached 90 μM. **(C)** Strong synergism was evident for most concentrations of gomisin B when ginsenoside Rh2 was 50.69 μM. **(D)** Expression of EGFR, ESR1, and MAPK1 in human neuroblastoma cells treated with gomisin B and ginsenoside Rh2.

The results of the study indicate that the viability of the four different NB cell lines was significantly reduced following incubation with Aidi injection, gomisin B, and ginsenoside Rh2 for 48 h. This reduction in cell viability was observed in cell cultures with 80% and 90% confluence, as well as fully confluent cell cultures. Furthermore, when compared to the control group, treatment of NB cells with gomisin B and ginsenoside Rh2 demonstrated a significant inhibition rate in a concentration-dependent manner. This suggests that the inhibitory effect on cell growth increases with increasing concentrations of gomisin B and ginsenoside Rh2 (Figure 7A). In contrast, Aidi injection did not exhibit a significant anti-tumor effect at the tested concentrations. The lack of significant inhibition of cell viability by Aidi injection suggests that it may not be effective in reducing the growth of these NB cells under the conditions tested. Notably, synergism, as indicated by a combination index of 0.43, was observed when gomisin B reached a GI_50_ value of 151.2 μM, and the concentration of ginsenoside Rh2 reached 90 μM (Figure 7B). Similarly, when ginsenoside Rh2 was at 50.69 μM, strong synergism was observed for the majority of gomisin B concentrations (Figure 7C). More details are shown in Supplementary Table S1.

### 3.10 Results of the effects of synergism or antagonism in gomisin B and ginsenoside Rh2 combination on cell viability

When the GI_50_ value of fixed gomisin B was 151.2 μM, no synergistic therapeutic effect of the two drugs was observed in the SK-N-SH cell line. This means that at this specific concentration of gomisin B, combining it with varying concentrations of ginsenoside Rh2 did not result in a greater inhibitory effect on cell growth compared to using ginsenoside Rh2 alone. On the other hand, when the GI_50_ value of fixed ginsenoside Rh2 was 50.69 μM, it could be seen that the two drugs had a good synergistic therapeutic effect. This suggests that at this particular concentration of ginsenoside Rh2, combining it with varying concentrations of gomisin B led to a significantly enhanced inhibitory effect on cell growth, indicating a positive synergistic interaction between the two drugs. The original data concerning the results of the effects of synergism or antagonism in gomisin B and ginsenoside Rh2 combination on NB cell line viability are shown in Table 3. Furthermore, more details are shown in Supplementary Table S2.

**TABLE 3:** Effects of synergism or antagonism in gomisin B and ginsenoside Rh2 combination on cell viability. (1) CI < 0.1, very strong synergism, +++++; (2) 0.1 < CI < 0.3, strong synergism, ++++; (3) 0.3 < CI < 0.7, synergism, +++; (4) 0.7 < CI < 0.85, moderate synergism, ++; (5) 0.85 < CI < 0.90, slight synergism, +; (6) 0.90 < CI < 1.10, nearly additive, ±; (7) 1.10 < CI < 1.20, slight antagonism, -; (8) 1.20 < CI < 1.45, moderate antagonism, --; (9) 1.45 < CI < 3.3, antagonism, ---; (10) 3.3 < CI < 10, strong antagonism, ----; (11) < 10, very strong antagonism, -----.

	Concentration (μM)	Combination index	Graded symbol
Gomisin B GI_50_ (151.2 μM) + ginsenoside Rh2	90	0.43	+++
	45	1.03	±
	22.5	0.95	±
	11.25	0.91	±
	5.625	0.91	±
	2.813	0.91	±
	1.406	0.89	+
Ginsenoside Rh2 GI_50_ (50.69 μM) + gomisin B	270	0.72	++
	135	0.89	+
	67.5	0.74	++
	33.75	0.51	+++
	16.875	0.41	+++
	8.439	0.35	+++
	4.219	0.32	+++

### 3.11 Results of the effects of gomisin B and ginsenoside Rh2 on potential targets *in vitro*

To corroborate the findings from NP analysis, SK-N-BE (2) and SH-SY5Y human NB cells were selected for experimental validation. The results of Western blot analysis demonstrated a significant increase in the protein level of ESR1 induced by gomisin B, particularly in MYCN-amplified NB cells (Figure 7D). Notably, there were no discernible differences in MAPK1 levels between both cell lines. The *in vitro* experimental outcomes aligned with the predictions derived from NP, suggesting that gomisin B might be the primary active compound in Aidi injection for NB treatment, especially for patients with MYCN-amplified NB.

## 4 Discussion

NB is the most common malignant exocranial solid tumor in children, which accounts for approximately 15% of the mortality of children due to malignant tumors (Maris, 2010). It is noted that TCM formulas exert effects through multiple targets, pathways, and BPs (Wang et al., 2020). It has been reported that Aidi injection is the most competitive product in cancer care in China, particularly used in NB treatment, and has shown a productive anti-tumor effect (Yang et al., 2022). However, its potential anti-tumor mechanisms remain unclear. This study constructed the relationships between the active components, targets, pathways, BPs, and diseases to investigate the underlying mechanisms of Aidi for NB treatment.

NP usually takes oral bioavailability (OB) ≥30%, and drug-likeness (DL) ≥0.18 as a compound selection strategy (Ru et al., 2014). The drug–compound–hub target gene–pathway network exhibited that 42 active compounds in Aidi involving 16 hub targets and 10 most relevant pathways were identified. Among the 42 active compounds, isoflavanone, gomisin B, and ginsenoside Rh2 were the most significant compounds with high degree values in the network. Further analysis using molecular docking showed that gomisin B and ginsenoside Rh2 were the key compounds of Aidi, which showed the greatest binding interactions with the target proteins associated with the survival of NB patients (Figure 5). Gomisin B was isolated from *Schisandra grandiflora*, a plant traditionally used in different TCM formulas (Poornima et al., 2017). A previous study investigated the anti-tumor ability of gomisin B and revealed its cytotoxic activities against six human cancer cells: SIHA (cervical cancer), PANC1 (pancreatic carcinoma), MDAMB-231 (breast cancer), IMR32 (neuroblastoma), DU-145 (prostate cancer), and A-549 (lung cancer) (Ogunsina et al., 2020). Ginsenoside Rh2, which was derived from *Panax ginseng* Meyer, exhibits anticancer activity in various human cancer cell lines both *in vitro* and *in vivo* by modulating several signaling pathways, such as those of PDZ-binding kinase/T-LAK cell-originated protein kinase, phosphatidylinositol 3-kinase, protein kinase B, mammalian target of rapamycin, epidermal growth factor receptor, p53, and reactive oxygen species. Ginsenoside Rh2 could induce NB cell apoptosis by activating caspase-1 and -3 and upregulating Bax, while p53 plays a significant role in the pathways to promote apoptosis (He et al., 2021; Zhang et al., 2021).

Our results from the final network highlighted 16 hub genes, of which only MAPK1, EGFR, and ESR1 were identified as important hub genes. MAPK1 is a serine–threonine kinase that belongs to the MAPK family, which is implicated in numerous cellular processes, such as cell cycle, cell apoptosis, and cell survival (Zhang et al., 2020). The literature has previously reported minimal expression of EGFR proteins in several NB cell lines (Zheng et al., 2016). Similarly, it has been revealed that ESR1 plays an important role in sympathetic nervous system development (Mirkin et al., 2002). Contrary to our expectations, our study identified that only gomisin B in Aidi injection effectively regulates ESR1 in MYCN-amplified NB cells. This finding aligns with the findings of Loven et al. (2010), suggesting that gomisin B may remodel the ESR1 expression. Additionally, our bioinformatic analyses of microarray data from NB tumors unveiled a correlation between high ESR1 expression and enhanced event-free survival in NB patients, indicating a favorable disease outcome (refer to Figure 3N).

Aidi displayed a close relationship with gland development, immune response-activating signal transduction, and immune response-activating cell surface receptor signaling pathways in the GO enrichment analysis. More than 90% of NB tumors arise in the adrenal gland, suggesting a link between perinatal tumors and adrenal development (Nuchtern, 2006). The findings of the KEGG pathway annotation indicated that Aidi might exert therapeutic effects mainly through endocrine resistance and estrogen signaling pathways.

In this study, gomisin B was the sole compound identified in Aidi injection, a finding substantiated through molecular docking and *in vitro* experiments. Nevertheless, certain limitations exist in our research. First, our investigation forecasted and confirmed the molecular mechanisms of Aidi for NB treatment at the system level, lacking *in vivo* evidence for validation. Although the study suggests encouraging anti-tumor effects of gomisin B in MYCN-amplified NB cells, further exploration is required to understand its actual mechanism in regulating ESR1 and its interactions with chemotherapy drugs.

## 5 Conclusion

Our research shows that gomisin B might be the main active compound in Aidi injection, and the protein target ESR1 may be a potential therapeutic target of Aidi injection in the treatment of NB. The study suggests that Aidi injection may exert anti-tumor effects by influencing endocrine resistance and estrogen signaling pathways. The findings highlight the complex involvement of various compounds, targets, BPs, and signaling pathways in the pharmacological mechanisms of Aidi for NB treatment. This research provides a foundation for further exploration in this area.

## Data Availability

The raw data supporting the conclusion of this article will be made available by the authors without undue reservation.
